# Analysis of the NCR Mechanisms in *Hanseniaspora vineae* and *Saccharomyces cerevisiae* During Winemaking

**DOI:** 10.3389/fgene.2018.00747

**Published:** 2019-01-11

**Authors:** Jessica Lleixà, Valentina Martín, Facundo Giorello, Maria C. Portillo, Francisco Carrau, Gemma Beltran, Albert Mas

**Affiliations:** ^1^Departament de Bioquímica i Biotecnologia, Facultat d’Enologia, Universitat Rovira i Virgili, Tarragona, Spain; ^2^Sección Enología, Food Science and Technology Department, Facultad de Química, Universidad de la República (UdelaR), Montevideo, Uruguay

**Keywords:** non-*Saccharomyces*, yeast assimilable nitrogen, nitrogen consumption, alcoholic fermentation, amino acids

## Abstract

There is increasing interest in the use of non-*Saccharomyces* yeasts in winemaking due to their positive attributes. The non-*Saccharomyces* yeast *Hanseniaspora vineae* is an apiculate yeast that has been associated with the production of wine with good fermentation capacity and an increase in aromatic properties. However, this yeast represents a concern in mixed culture fermentation because of its nutrient consumption, especially nitrogen, as its mechanisms of regulation and consumption are still unknown. In this study, we analyzed the nitrogen consumption, as well as the nitrogen catabolism repression (NCR) mechanism, in two genome-sequenced *H. vineae* strains, using synthetic must fermentations. The use of synthetic must with an established nitrogen content allowed us to study the NCR mechanism in *H. vineae*, following the amino acid and ammonia consumption, and the expression of genes known to be regulated by the NCR mechanism in *S. cerevisiae*, *AGP1*, *GAP1*, *MEP2*, and *PUT2*. *H. vineae* exhibited a similar amino acid consumption and gene expression profile to *S. cerevisiae*. However, the wine strain of *S. cerevisiae* QA23 consumed ammonia and valine more quickly and, in contrast, tyrosine and tryptophan more slowly, than the *H. vineae* strains. Our results showed a similar behavior of nitrogen regulation in *H. vineae* and *S. cerevisiae*, indicating the presence of the NCR mechanism in this *Hanseniaspora* yeast differentiated before the whole genome duplication event of the *Saccharomyces* complex. Future study will elucidate if the NCR mechanism is the only strategy used by *H. vineae* to optimize nitrogen consumption.

## Introduction

For many years, the microbiological process of winemaking has been focused on the use of starter cultures of *Saccharomyces cerevisiae*. The inoculation of commercial strains of *S. cerevisiae* is a common practice in wineries to ensure the completion of the fermentation and the quality of the final product. However, the elaboration of uniformed wines is not always desired, and winemakers are becoming more interested in obtaining characteristic and differential wines. Considering this fact, in recent years, much effort has been focused on the use of non-*Saccharomyces* yeasts to obtain wine with new organoleptic characteristics (Fleet, 2008; Jolly et al., 2014; Carrau et al., 2015). Non-*Saccharomyces* yeasts are naturally present on grape surfaces, and they can start spontaneous fermentations that can lead in incomplete fermentations or result in wines with unpleasant properties. Despite this fact, many of these non-*Saccharomyces* yeasts have proven to produce enzymatic activities and release metabolites that improve some oenological processes and the wine flavor (Jolly et al., 2014; Padilla et al., 2016; Varela, 2016). For this reason, the interest in the use of co-fermented or sequential mixed cultures of non-*Saccharomyces* and *S. cerevisiae* has increased to take advantage of both trends during the winemaking process.

*Hanseniaspora vineae* is one species of yeast that belongs to the non-*Saccharomyces* yeasts of oenological interest (Martin et al., 2018). The primary positive contributions of this yeast during the winemaking process are basically related to the aroma profile in the final wine. *H. vineae* has been demonstrated to increase fruity aromas and produce high amounts of acetate esters, primarily 2-phenylethyl acetate and benzenoids, in wines elaborated in either synthetic (Martin et al., 2016) or natural musts inoculated with *H. vineae* (Lleixà et al., 2016) or by sequential fermentation with *S. cerevisiae* (Viana et al., 2011; Medina et al., 2013). The higher ester content produced by this non-*Saccharomyces* yeast can be explained by its prominent *β*-glucosidase activity that enables it to release these compounds into the media (Barquet et al., 2012; López et al., 2015).

The development of non-*Saccharomyces* yeasts can affect the growth of the primary wine yeast *S. cerevisiae* and the fermentation progress as a consequence of the consumption of important nutrients, such as nitrogen and vitamins (Medina et al., 2012). Some studies confirmed the effect of non-*Saccharomyces* yeasts on nutrient availability in mixed cultures. Andorrà et al. (2010) observed that mixed cultures with *Candida zemplinina* and *Hanseniaspora uvarum* had a higher amino acid consumption than pure cultures of these yeasts. Indeed, pure and mixed cultures showed a preferential uptake of some amino acid groups related with the synthesis of aroma compounds that might be strain-dependent as was shown for *S. cerevisiae* (Bisson, 1991). This higher nitrogen consumption also happened in mixed cultures with *H. vineae* (Medina et al., 2012). The moment of inoculation, simultaneous or sequential, and the inoculum size in mixed cultures determine the progress of the fermentation because of the nutrient competition between the *Saccharomyces* and non-*Saccharomyces* yeasts. Some researchers have demonstrated that a sequential fermentation resulted in sluggish or stuck fermentations as a consequence of the nutrient consumption of the non-*Saccharomyces* strain, which reduced the nutrient availability to the *Saccharomyces* strain (Medina et al., 2012; Taillandier et al., 2014). In *S. cerevisiae* has been observed to activate the genes responsible for nitrogen and glucose metabolism to prevent this situation when it was co-cultivated with different non-*Saccharomyces* yeast to decrease the nutrients available to the non-*Saccharomyces* yeast (Curiel et al., 2017). In addition, recent studies focused on the specific use of ammonia and amino acids by the different non-*Saccharomyces* species and its implication on *S. cerevisiae* performance in sequential fermentations. Non-*Saccharomyces* yeast have been shown to exhibit a specific amino acid consumption profile depending on the yeast species, which interferes with *S. cerevisiae* development and generates changes in the volatile profile during sequential fermentations (Gobert et al., 2017; Rollero et al., 2018). In summary, the specific nutrient addition of amino acids, ammonia or vitamins has to be evaluated to ensure a good fermentation performance under sequential yeast inoculation (Medina et al., 2012; Gobert et al., 2017; Rollero et al., 2018).

Specifically, in grape must, we can find different nitrogen compounds, but only some of them can be consumed by *S. cerevisiae* to produce biomass and encourage the fermentation process. These compounds, known as Yeast Assimilable Nitrogen (YAN), are comprised of the ammonia and the amino acids present in the grape juice (Bell and Henschke, 2005). From this YAN, we can differentiate the preferred nitrogen sources, such as ammonia, asparagine, and glutamine, which promote *S. cerevisiae* growth, and the non-preferred nitrogen sources, such as urea, that result in a low growth rate when it grows only with those nitrogen sources (Ter Schure et al., 2000; Magasanik and Kaiser, 2002).

Therefore, *S. cerevisiae* has developed a mechanism called Nitrogen Catabolism Repression (NCR) that selects the best nitrogen sources for growth. The NCR mechanism consists in the reduction of proteins responsible for utilization and uptake of non-preferred nitrogen sources in the presence of preferred nitrogen sources. This mechanism acts at two levels to assure the consumption of preferred nitrogen sources. The first consists in the inactivation and degradation of the existing non-preferred nitrogen source permeases, and the second consists in the repression of genes encoding for non-preferred nitrogen source permeases (Ter Schure et al., 2000; Magasanik and Kaiser, 2002).

From the 19 amino acid permeases that *S. cerevisiae* contains, there are three high-capacity permeases that are nitrogen-regulated, including *AGP1* (high-Affinity Glutamine Permease), *GAP1* (General Amino acid Permease) and *PUT4* (Proline UTilization). In addition, other non-permeases proteins like *PUT2* (Delta-1-pyrroline-5-carboxylate dehydrogenase), which is a key enzyme for the conversion of proline into glutamate in the mitochondria once it has entered the cell through *PUT4*, are also nitrogen-regulated (Hofman-Bang, 1999). The amino acid permesases *GAP1* and *PUT4* together with the dehydrogenase *PUT2* are active during growth in non-preferred nitrogen content and repressed in the presence of a preferred nitrogen source, such as ammonium (Forsberg and Ljungdahl, 2001). Alternatively, *AGP1* is active in the presence of a preferred nitrogen source and repressed when this nitrogen is consumed (Regenberg et al., 1999).

In the case of ammonium, three permeases are responsible of its uptake, namely *MEP1*, *MEP2*, and *MEP3*. When the concentration of ammonia in the medium is low, these permeases become active. However, in a non-preferred nitrogen source, the expression of *MEP2* is much higher than those of *MEP1* and *MEP3*, since it is the one with the highest affinity for ammonium (Ter Schure et al., 2000). Previous studies have reported that the expression of those nitrogen-regulated proteins can be used as a biomarker for nitrogen deficiency in wine fermentations (Beltran et al., 2005; Deed et al., 2011; Gutiérrez et al., 2013). The study of the expression of these proteins can be also an indirect evidence of the existence of NCR mechanism. In fact, NCR genes are regulated by several transcription factors, amongst others Gln3 and Nil1, and also by their regulator Ure2p. Under nitrogen limitation, Gln3 dissociates from Ure2p, the dephosphorylated Gln3 goes to the nucleus and increases the transcription of genes containing UAS_NTR_ sequence (Upstream activating sequence), like *GAP1*, *PUT4, PUT2*, and *MEP2* genes (Ter Schure et al., 2000; Tesnière et al., 2015).

Nitrogen metabolism and the NCR mechanism have been deeply studied in *S. cerevisiae*, both in laboratory and wild strains, showing the multiple mechanisms used by this species under nitrogen-limited conditions (Beltran et al., 2004; Godard et al., 2007; Gutiérrez et al., 2013; Tesnière et al., 2015). However, very little is known about nitrogen preferences and regulation in non-*Saccharomyces* species. A better understanding of nitrogen utilization among the different yeast species is important to increase the efficiency, predictability and quality of wine production, as well as of other biotechnological uses of yeast. The great variability on respiro-fermentative metabolism observed in non-*Saccharomyces* yeasts (Gonzalez et al., 2013) is an example of the possible divergences in nitrogen metabolism between *Saccharomyces* and non-*Saccharomyces* species. One of the limitations for performing molecular studies on non-*Saccharomyces* yeasts has been the lack of genomic data. Fortunately, in the last decade, the genomes of a large number of wine yeast species have been sequenced (Masneuf-Pomarede et al., 2016), and these sequences are available for molecular or genetic studies, such as those of the wine yeast *H. vineae* (Giorello et al., 2014).

In summary, the use of non-*Saccharomyces* yeasts is increasing to produce new wine styles taking advantage of their potential abilities. The nitrogen availability is important for yeast for its growth, as well as for the production of volatile compounds during the fermentation process. The mechanism used by *S. cerevisiae* to select the best nitrogen source is well known and documented, while it has not been studied in non-*Saccharomyces* yeasts.

The aim of this study was to evaluate the presence of the nitrogen catabolite repression (NCR) mechanism in *H. vineae*. We performed laboratory-scale fermentations of *H. vineae* and *S. cerevisiae* using a synthetic must with a defined nitrogen content. We followed the expression of the ortholog NCR-sensitive genes in *H. vineae* and the amino acid and ammonium consumption during the fermentation. Finally, we compared the results of *H. vineae* fermentations with the fermentations performed using a commercial *S. cerevisiae* strain.

## Materials and Methods

### Yeast Strains

The commercial wine yeast strain used in this study was *Saccharomyces cerevisiae* QA23 (Lallemand^®^, Canada). The apiculate yeast strains used, *Hanseniaspora vineae* T02/5AF and *Hanseniaspora vineae* T02/19AF, were both isolated from Uruguayan vineyards (Barquet et al., 2012). The use of two strains of *H. vineae* responds to the need of validating the results in this specie since the strains chosen have shown differences in aroma production which could be related with nitrogen metabolism (Martin et al., 2016).

Yeast strain *S. cerevisiae* QA23 was in active dry yeast (ADY) form. The rehydration process was performed according to the manufacturer’s instructions (Lallemand^®^, Canada). Both strains of *H. vineae*, T02/5AF and T02/19AF, were in fresh paste form, and both were prepared in the same way as QA23 using warm water.

### Fermentation Conditions

To determine the uptake and metabolism of nitrogen, yeast strains were grown at 28°C during 24 h in a solid yeast extract-peptone dextrose (YPD) medium (1% yeast extract, 2% peptone, 2% glucose, and 1.7% agar). A colony from the yeast culture was inoculated in 50 mL liquid YPD media for 24 h in Erlenmeyer flasks at 120 rpm and 28°C. A population of 1 × 10^6^ cells/mL of the yeast strain was inoculated into an Erlenmeyer flask with 100 mL of yeast nitrogen base (YNB) media without amino acids (Difco^TM^) with 150 mg/L of (NH_4_)SO_4_ and 20 g/L of glucose (AppliChem Panreac^®^) for 24 h at 120 rpm and 28°C. The YNB medium was used to exhaust the yeast nitrogen reserves.

After a microscopic counting of the cells using a Neubauer chamber, 1,500 mL of synthetic must was inoculated to a final concentration of 2 × 10^6^ cells/mL. The cells were washed and resuspended with synthetic must before inoculation to remove the nitrogen residues.

The fermentations were performed in synthetic must (Supplementary Table S1) with a nitrogen content of 140 mg YAN/L (Supplementary Table S2) since this concentration has been established as the ideal one to achieve a complete fermentation without residual or excess nitrogen (Bely et al., 1990).

The fermentations were conducted in triplicate in laboratory-scale fermenters, i.e., 500 mL bottles filled with 440 mL of synthetic must and covered with a cap with two tubes that allowed sampling and the exit of carbon dioxide. The fermenters were maintained on a rotating shaker at 120 rpm at room temperature (22–23°C). The fermentation activity was assayed by the juice density every day using a portable density meter (Mettler Toledo).

### Cell Growth Measurements

In the laboratory-scale fermentations, cell population monitoring was established by measuring the absorbance at 600 nm. The samples were measured every 4 h during the first 48 h after inoculation and once a day from 48 h to the end of the fermentation.

### Determination of Relative Gene Expression

The evaluation of the gene expression affected by the nitrogen catabolite repression (NCR) was performed during the first hours on the synthetic must fermentation. Sampling every 4 h during the first 24 h and every 6 h from 24 to 36 h was followed by centrifugation (16,000 rpm, 5 min and 4°C) and removal of the supernatant. The pellet was washed with cold sterile MilliQ water (Millipore Q-POD^TM^ Advantage A10), centrifuged (16,000 rpm, 5 min, and 4°C) and after removal of the supernatant, it was frozen in liquid nitrogen and stored at −80°C.

The RNA was extracted from these samples using an RNeasy^®^ Mini kit (QIAGEN^®^) and RNase-Free DNAse Set (QIAGEN^®^) according to the manufacturer’s instructions. The RNA obtained was then measured using a Nano Drop (NanoDrop 1000 Thermo^®^ Scientific) and diluted to a final concentration of 320 ng/μL in a total volume of 11 μL. The cDNA synthesis of each sample was performed using the corresponding RNA, 1 μL of oligo-dT primer (Invitrogen^TM^), 1 μL of dNTPs (10 mM) and 1 μL of transcriptase (SuperScript^®^ II Reverse Transcriptase-Invitrogen^TM^) and amplified using a 2720 Thermal Cycler (Applied Biosystems) according to the manufacturer’s instructions.

The genes evaluated in this experiment considering their role in the NCR mechanism were *AGP1*, *GAP1*, *MEP2*, and *PUT2* and their orthologous in *H. vineae.* Annotation of putative orthologous was based on BLASTx searches using *H. vineae* predicted CDS and the proteome of *S. cerevisiae*. A hit was considered significant if: (i) e-value threshold was less than 1e-10 (ii) the alignment length covered more than 90% of the length of both sequences, and (iii) both sequences presented the same pfam domain. In case of multiple hits we selected the *H. vineae* prediction with higher percentage of amino acid identity (Supplementary Table S3). Primer design for each gene was performed using Primer Express software (Primer Express 3.0 Applied Biosystems) (Table 1). The housekeeping genes encoding Actin (*ACT1*) and Inorganic PyroPhosphatase 1 (*IPP1*) from *S. cerevisiae* and *H. vineae* were used to normalize the amplification curves of the selected genes considering their stability (Ståhlberg et al., 2008). All samples from each fermentation replicate were analyzed in duplicate.

**Table 1:** Primers used for the analysis of the expression of NCR-related genes in *H. vineae*.

Gene	Name	Oligonucleotide sequence (5′–3′ end)
*ACT1*	ACT-F	GGCTTCTTTGACCACTTTCCAA
	ACT-R	GATGGACCACTTTCGTCGTATTC
*AGP1*	AGP1-F	ATTGCTGGGTGACGGTTCTT
	AGP1-R	TGACATTGGTAGCGGCAATAAC
*GAP1*	GAP1-F	CAAAGGTTTGCCATCTGTCATC
	GAP1-R	TGCAGAGTTACCGACAGACAACA
*MEP2*	MEP2-F	TCGATGACGGGTTGGATGTT
	MEP2-R	CATAACGGCACCACTTAAACCA
*PUT2*	PUT2-F	GATACGACATGTTGGCAGCAA
	PUT2-R	TTTCGCCTTGGAAAACGTTT

*The primer design for the H. vineae genes was performed using Primer Express software (Primer Express 3.0 Applied Biosystems). The primers used for S. cerevisiae have been previously described by Beltran et al. (2004) and Gutiérrez et al. (2013).*

In all the samples, the Real-Time Quantitative PCR reaction was performed using 10 μL of SYBR Green [SYBR^®^ Premix Ex Taq II (Tli RNaseH Plus)], 0.4 μL of ROX Reference Dye (SYBR^®^ Premix Ex Taq II), 0.8 μL of each specific primer (10 μM) and 6 μL of sterile MilliQ water (Millipore Q-PODTM Advantage A10). The amplification process was conducted using a 7300 Real Time PCR System (Applied Biosystems) as follows: 50°C for 2 min, 95°C for 10 min and 40 cycles at 95°C for 15 s, 60°C for 2 min and 72°C for 30 s.

The relative gene expression was determined using the 2^−ΔΔ*C*_t_^ method (Beltran et al., 2005), where the *C*_t_ value corresponds to the number of cycles needed to achieve the background fluorescence. This method is used to compare the *C*_t_ values of the gene of interest, and the *C*_t_ values of the reference genes (*ACT1* and *IPP1*) (Δ*C*_t_); and −ΔΔ*C*_t_ consists of the difference of Δ*C*_t_ from the samples of each time point, and the Δ*C*_t_ of the reference time (4 h after inoculation). Results were expressed as the mean Log10 relative gene expression. All samples were analyzed in triplicate, and the resulting Log10 2^−ΔΔ*C*_t_^ values were statistically analyzed using ANOVA and Tukey’s post-test.

### Nitrogen Content Analysis of Laboratory Fermentation

The individual amino acid and ammonium contents of each sample were determined using high-performance liquid chromatography (HPLC) (Agilent 1100 Series HPLC) (Gómez-Alonso et al., 2007). The sample (400 μL) was mixed with borate buffer (700 μL), methanol (300 μL), diethyl ethoxymethylenemalonate (DEEM) (15 μL) and L-aminoadipic acid (internal control) (10 μL). After 2 h at 80°C, 50 μL of each sample was directly injected into the HPLC, which consists of a low pressure gradient quaternary pump, a thermostatted autosampler, a DAD ultraviolet detector and a fluorescence detector (Agilent Technologies, Germany). The separation process of the sample was performed using a 4.6 × 250 mm × 5 μm Hypersil ODS column (Agilent Technologies, Germany).

The solvent system was as follows: A solvent (mobile phase) [4.1 g of sodium acetate anhydrous diluted in 250 mL of MilliQ water, adjusted to pH 5.8 with glacial acetic acid and 0.4 g of sodium azide brought to a final volume of 2 L with MilliQ water (Millipore Q-POD^TM^ Advantage A10)] and B solvent (stationary phase) [80% acetonitrile and 20% methanol]. The analytical temperature was 20°C, and the flow rate was 0.9 mL/min. The concentration of each amino acid and ammonia was calculated using an external calibration curve of each component and expressed as mg N/L. The software used for the integration was Agilent ChemStation Plus (Agilent Technologies, Germany).

### Statistical Analysis

Statistical analysis of the gene expression data was performed using an ANOVA and indicated by the Tukey’s post-test (all pair comparisons) using XLSTAT Software. The results were considered statistically significant at a *p*-value less than 0.05.

## Results

### Fermentation Kinetics and YAN Consumption

**FIGURE 1:**
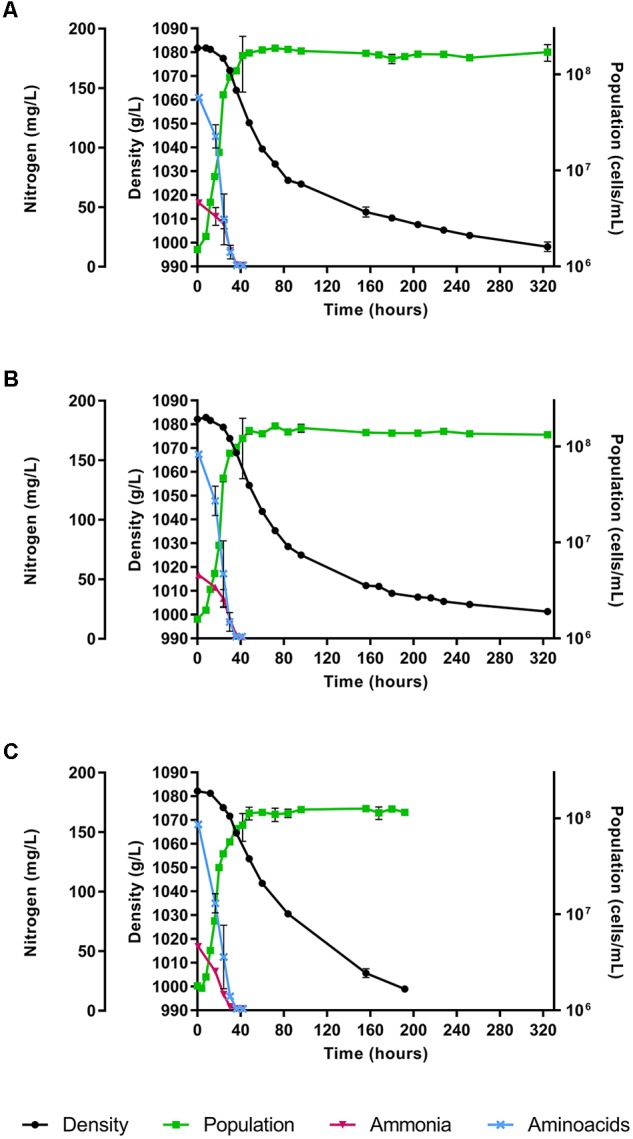
Fermentation kinetics, cell growth and YAN consumption expressed separately as amino acids and ammonia of **(A)** *Hanseniaspora vineae* T02/5AF. **(B)** *Hanseniaspora vineae* T02/19AF, and **(C)** *Saccharomyces cerevisiae* QA23 during alcoholic fermentation. The figure shows the mean of the three fermentation replicates feach strain and its standard deviation.

The fermentations were performed using a synthetic must with a nitrogen content of 140 mg YAN/L (corresponding to 190 mg N/L). Two strains of *H. vineae*, T02/5AF and T02/19AF, were evaluated, and *S. cerevisiae* strain QA23 was used as a control. Media density, cell growth and nitrogen content were assessed along with alcoholic fermentation. Both *H. vineae* strains showed a similar behavior in fermentation kinetics, cell growth and YAN consumption (Figure 1). These strains achieved a must density below 1000 g/L in approximately 13 days (324 h), while the *S. cerevisiae* strain was faster and reached this point in 8 days (192 h). The YAN was completely consumed by all the strains during the exponential growth phase that coincides with the initial stages of the fermentation (Figure 1). Ammonia and amino acids were consumed in 36 h by the *H. vineae* strains. Even though *S. cerevisiae* also consumed all the amino acids in 36 h, it exhausted the ammonia earlier, specifically before 30 h.

**FIGURE 2:**
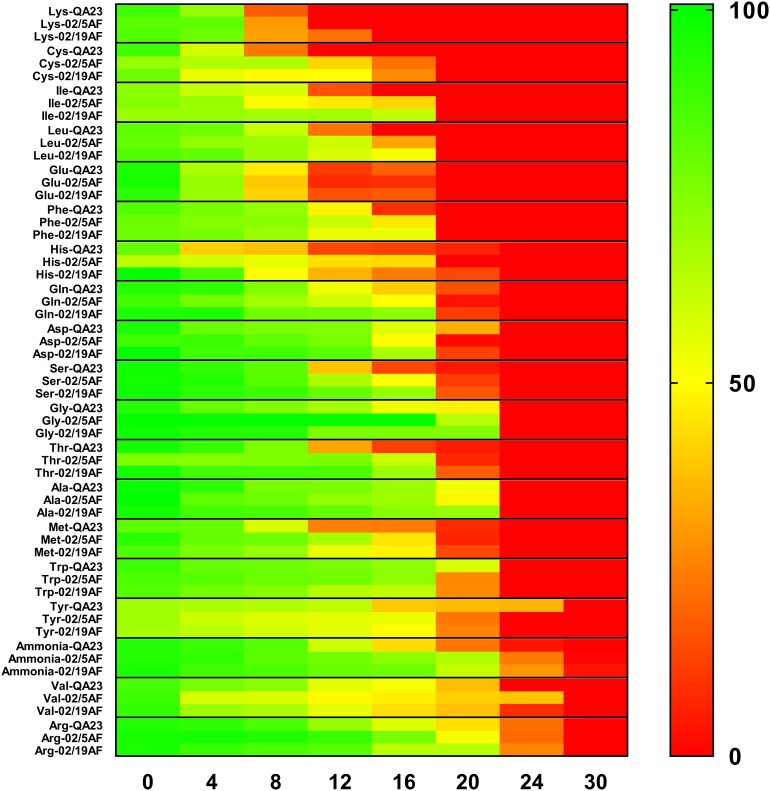
Heatmap of the available percentage of each amino acid in the must during the first 30 h of *H. vineae* T02/5AF, *H. vineae* T02/19AF and *S. cerevisiae* QA23 fermentations. Green color corresponds to 100% of the total amino acid content available, and red corresponds to 0% of amino acid content available in the media. Standard deviations were always lower than 10% and have been avoided in the figure for clarity.

The consumption of each amino acid and ammonia was measured during the first 36 h of the different strain fermentations. Figure 2 and Table 2 show the evolution of their consumption at different time points. In general, a similar consumption pattern of amino acids occurred in both *H. vineae* strains and *S. cerevisiae*. In all cases, lysine, glutamic acid, cysteine, isoleucine, leucine, and phenylalanine were completely assimilated during the first 24 h. As for the previous amino acids, histidine was also exhausted during this period solely by the *H. vineae* strain T02/5AF. The slowest consumed amino acids, arginine, and valine, were still available in very small amounts after 30 h in both *S. cerevisiae* and *H. vineae*. The remaining amino acids were consumed between 24 and 36 h in every case.

**Table 2:** Time (h) required for each yeast strain to exhaust the different nitrogen compounds of the synthetic must.

	Consumed in 16–20 h	Consumed in 20–24 h	Consumed in 24–30 h	Consumed in 30–36 h
*H. vineae* T02/5AF	Lys	Glu, His, Cys, Ile, Leu, Phe	Asp, Ser, Gln, Gly, Thr, Ala, Met, Trp, Tyr	Arg, Val, NH_4_^+^
*H. vineae* T02/19AF	Lys	Glu, Cys, Ile, Leu, Phe	Asp, Ser, Gln, His, Gly, Thr, Ala, Trp, Tyr, Met	Arg, Val, NH_4_^+^
*S. cerevisiae* QA23	Lys, Cys, Ile, Leu	Glu, Phe	Asp, Ser, Gln, His, Gly, Thr, Ala, Val, Met Trp, NH_4_^+^	Arg, Tyr

### Expression of NCR-Regulated Genes

Different genes related to NCR mechanism were evaluated for their homology in *H. vineae* including three permeases (*AGP1*, *GAP1*, and *MEP2*), one dehydrogenase (*PUT2)* and four transcriptional factors (*GAT1, GLN3, GZF3*, and *DAL80*). Except from *DAL80*, all the other genes had their homologous in *H. vineae* suggesting the presence of this nitrogen regulation mechanism in this yeast (Supplementary Table S3). To further check that this species displays this regulation, four genes related to nitrogen transport into the cell (*AGP1*, *GAP1*, and *MEP2*) and one gene related to proline utilization (*PUT2)* were selected to analyze their expression pattern during the first fermentation hours. These genes have been described and used in *S. cerevisiae* as markers for nitrogen limitation (Beltran et al., 2004, 2007; Gutiérrez et al., 2013) and also as an indirect marker of transcriptional factors activity.

**FIGURE 3:**
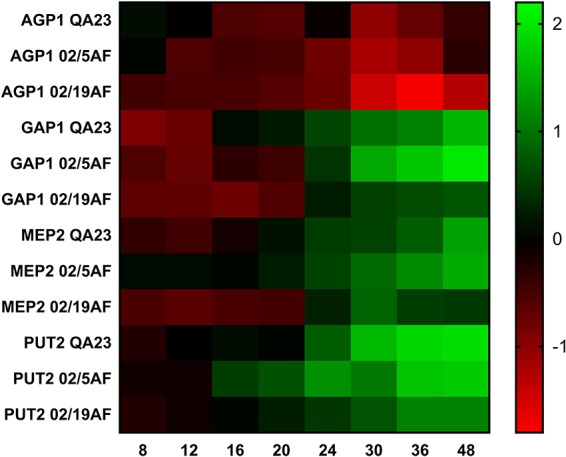
Heatmap of the expression of *AGP1, GAP1, MEP2* and *PUT2* at different time points during the first 48 h of the fermentation for each yeast strain. Green color indicates an activation of gene expression, while red color indicates the repression of gene expression. Standard deviations have been avoided in the figure for clarity.

Figure 3 and Supplementary Table S4 show the expression evolution of the different genes during the first 48 h for each strain. Gene expression at 4 h was considered to be a reference, since the expression at 0 h corresponds to the inoculum that was nitrogen-depleted. The pattern of gene expression was similar for all the strains. *AGP1* was the only gene that was down-regulated during the fermentation, compared to the expression obtained at 4 h. Thus, the expression of *AGP1* is higher at the beginning of the fermentation (e.g., 4 h, our reference time), when the amino acid concentration is also higher, and decreases as the amino acids are consumed. The other three genes started to be up-regulated at different time points along the fermentation depending on the yeast species. Therefore, both *H. vineae* strains activated the *GAP1* and *MEP2* expression after 24 h, and *PUT2* after 16 h of fermentation. Finally, *S. cerevisiae* QA23 expressed *GAP1*, *MEP2*, and *PUT2* after 16, 20 and 24 h of fermentation, respectively. Despite the differences between the yeast strains, the gradual activation or repression of the different genes coincided with the progressive consumption of the amino acids and ammonia during fermentation.

## Discussion

In this study, we aimed to determine if *H. vineae*, a non-*Saccharomyces* yeast of oenological interest, displays the NCR mechanism under fermentation conditions. This metabolism has been thoroughly studied in *S. cerevisiae* during alcoholic fermentation (Beltran et al., 2004; Tesnière et al., 2015), and it was considered to be a reference in this study. Our results suggest that *H. vineae* exhibits an NCR mechanism similar to that of *S. cerevisiae*.

Fermentations using synthetic must with 140 mg YAN/L allowed us to evaluate ammonia and amino acid consumption together with the analysis of the expression of NCR-regulated genes during the first hours of fermentation. First, the nitrogen content of the synthetic must used in this study was not limiting, and it is considered to be the minimum concentration needed for yeasts to complete the alcoholic fermentation (Ribéreau-Gayon et al., 2006). In fact, all the strains tested in this work were able to complete the fermentation process (Figure 1) which agrees on previous reports (Ribéreau-Gayon et al., 2006). However, as we expected, *S. cerevisiae* finished the fermentation more quickly, because of its oenological abilities to resist the fermentation conditions. In addition, *H. vineae*, as well as *S. cerevisiae*, exhausted all the available YAN in 36 h even though *S. cerevisiae* consumed all the ammonia in 30 h, 6 h sooner than *H. vineae* (Figure 2 and Table 2). Medina et al. (2012) showed a competition for nutrients, especially nitrogen, in mixed fermentations of *S. cerevisiae* and *H. vineae*. The similar consumption of the nitrogen of these two yeasts observed in this study would explain the competition for this nutrient noted by Medina et al. (2012) in mixed fermentations.

In the same way as previous studies performed in *S. cerevisiae*, the strains evaluated exhausted all the YAN during the growth phase demonstrating how nitrogen availability plays a role as a limiting fermentation factor (Beltran et al., 2004; Crépin et al., 2012). In addition, the kinetic consumption of different nitrogen compounds has been evaluated simulating oenological conditions in different *S. cerevisiae* strains and conditions in different studies. Beltran et al. (2007) demonstrated how temperature affects the amino acid intake, which affects yeast growth and metabolism. In our case, the fermentations proceeded at 22–23°C, and the consumption pattern of the nitrogen compounds was similar to that reported by Crépin et al. (2012). In fact, Crépin et al. (2012) classified nitrogen compounds in three groups according to their order of use by different *S. cerevisiae* strains: prematurely consumed, early consumed and late consumed. We classified the nitrogen compounds considering the time it took for them to be completely exhausted by each yeast strain (Table 2). However, we can observe that lysine is the fastest to be consumed by all the strains, and it is the one classified as prematurely consumed or that arginine, valine, tyrosine, and NH_4_^+^ are the later ones to be completely exhausted, which belong to the late consumed compounds group established by Crépin et al. (2012). Considering these aspects, we observed that *H. vineae* has a similar behavior to *S. cerevisiae* in nitrogen uptake, and the variability of nitrogen compound preferences in *H. vineae* appear to also depend on the strain coinciding with previous studies on different *S. cerevisiae* strains (Crépin et al., 2014).

Interestingly, arginine was the slowest amino acid to be consumed in all cases. This amino acid is known as a non-preferred nitrogen source, since its support to yeast growth is very poor (Cooper, 1982), and it is the most stored amino acid in the vacuole during the growth phase (Crépin et al., 2014). In addition, the evaluation of arginase activity was proposed to be an indicator of the available nitrogen in fermentation (Carrasco et al., 2003), because as nitrogen becomes limiting, yeasts start to metabolize the stored nitrogen for additional growth (Crépin et al., 2014). In addition, Beltran et al. (2004) observed that the activation of arginase activity coincides with the mobilization of arginine, the ammonium depletion and the activation of *GAP1*. In this study, the highest arginine consumption coincided with ammonia depletion in *S. cerevisiae*. However, in *H. vineae*, arginine intake is simultaneous to that of ammonium, which may indicate that this yeast species uses a different way to store or consume this amino acid. The lower preference of *H. vineae* for ammonium is consistent with the reported poor effect of ammonium addition to agave juice fermentations compared to other nitrogen sources (Díaz-Montaño et al., 2010). In addition, *H. vineae* strains produced significantly lower levels of isobutyl alcohol derived from valine (Martín, 2016), which could be related to the slower consumption of this amino acid exhibited by this yeast species in this study.

The gene expression of *GAP1, MEP2*, and *PUT2* evolved from nitrogen-repressed to nitrogen-activated conditions as nitrogen was consumed in all cases. Between 16 and 30 h after inoculation with the different yeast strains, the gene expression of *GAP1, MEP2*, and *PUT2* began to be significantly activated. On the other hand, *AGP1* began to be repressed after 8 and 12 h of fermentations. As described before, *AGP1* acts as a sensor for amino acids, and its expression is induced by extracellular amino acids via SPS system, and down-regulated when the amino acids are consumed (Regenberg et al., 1999; Godard et al., 2007), which is consistent with our results. In the case of *GAP1* and *PUT2*, the transcription of these genes is known to be activated under limiting nitrogen conditions (Forsberg and Ljungdahl, 2001; Gutiérrez et al., 2013), and this fact would explain their upregulation once the most preferred nitrogen compounds are consumed. Finally, we analyzed the ammonium permease *MEP2* expression, which is notably higher than other ammonium permeases (Ter Schure et al., 2000). Previous studies in *S. cerevisiae* have observed the activation of both *GAP1* and *MEP2* when ammonium is depleted (Beltran et al., 2004, 2005). However, in our study, the three strains tested showed a gradual activation of these two genes as ammonium and preferred amino acids were being consumed. From these results, we can deduce the activation of the transcriptional factors responsible for the expression of NCR genes.

The homology found on the NCR related proteins between *H. vineae* and *S. cerevisiae*, as well as the similarity in nitrogen consumption and the regulation of NCR genes, suggested the presence of the NCR mechanism in this non-*Saccharomyces* yeast. In addition, similarly to what has been described in *S. cerevisiae* (Beltran et al., 2004; Tesnière et al., 2015), the *H. vineae* wine yeasts evaluated entered the stationary phase coinciding with the exhaustion of nitrogen and consequently, the upregulation of the NCR genes. However, further research would be necessary to fully understand nitrogen metabolism in *H. vineae* and to elucidate if other mechanisms not regulated by NCR are responsible for nitrogen transport in this yeast.

Finally, the aim of this study was to determine if *H. vineae*, a non-*Saccharomyces* yeast of oenological interest, exhibits the NCR mechanism. Since nitrogen is one of the most limiting factors during alcoholic fermentation, knowing how it is metabolized gains importance. For that reason, we performed fermentations using synthetic must with an established nitrogen content, and we analyzed the nitrogen consumption and the expression of the NCR-regulated genes. The observed pattern of gene expression and nitrogen intake for the *H. vineae* strains and *S. cerevisiae* was similar, suggesting the presence of this regulatory mechanism in *H. vineae*. This study contributes to a better understanding of nitrogen metabolism in the most active species in terms of the fermentation capacity of the genus *Hanseniaspora*, yeasts differentiated before the whole genome duplication event of the *Saccharomyces* group. In addition to our results, more studies are needed to completely understand nitrogen metabolism in this species.

## Author Contributions

JL performed and designed the experiments, wrote the manuscript, and discussed and analyzed the results. VM, FG, and FC analyzed and discussed the results. MP analyzed and discussed the results, and wrote the manuscript. GB and AM designed the experiments, analyzed and discussed the results, and wrote the manuscript.

## Conflict of Interest Statement

The authors declare that the research was conducted in the absence of any commercial or financial relationships that could be construed as a potential conflict of interest.

## References

[B1] Andorrà I. Berradre M. Rozès N. Mas A. Guillamón J. M. Esteve-Zarzoso B. (2010). Effect of pure and mixed cultures of the main wine yeast species on grape must fermentations. *Eur. Food Res. Technol.* 231 215–224. 10.1007/s00217-010-1272-0

[B2] Barquet M. Martín V. Medina K. Pérez G. Carrau F. Gaggero C. (2012). Tandem repeat-tRNA (TRtRNA) PCR method for the molecular typing of non-*Saccharomyces* subspecies. *Appl. Microbiol. Biotechnol.* 93 807–814. 10.1007/s00253-011-3714-4 22113560

[B3] Bell S.-J. Henschke P. A. (2005). Implications of nitrogen nutrition for grapes, fermentation and wine. *Aust. J. Grape Wine Res.* 11 242–295. 10.1111/j.1755-0238.2005.tb00028.x

[B4] Beltran G. Esteve-Zarzoso B. Rozès N. Mas A. Guillamón J. M. (2005). Influence of the timing of nitrogen additions during synthetic grape must fermentations on fermentation kinetics and nitrogen consumption. *J. Agric. Food Chem.* 53 996–1002. 10.1021/jf0487001 15713011

[B5] Beltran G. Novo M. Rozès N. Mas A. Guillamón J. M. (2004). Nitrogen catabolite repression in *Saccharomyces cerevisiae* during wine fermentations. *FEMS Yeast Res.* 4 625–632. 10.1016/j.femsyr.2003.12.004 15040951

[B6] Beltran G. Rozès N. Mas A. Guillamón J. M. (2007). Effect of low-temperature fermentation on yeast nitrogen metabolism. *World J. Microbiol. Biotechnol.* 23 809–815. 10.1007/s11274-006-9302-6

[B7] Bely M. Sablayrolles J. M. Barre P. (1990). Automatic detection of assimilable nitrogen deficiencies during alcoholic fermentations in enological conditions. *J. Ferment. Bioeng.* 70 246–252. 10.1016/0922-338X(90)90057-4

[B8] Bisson L. F. (1991). “Influence of nitrogen on yeast and fermentation of grapes,” in *Proceedings of the International Symposium on Nitrogen in Grapes and Wine*, Seattle, 78–79.

[B9] Carrasco P. Pérez-Ortín J. E. Del Olmo M. (2003). Arginase activity is a useful marker of nitrogen limitation during alcoholic fermentations. *Syst. Appl. Microbiol.* 26 471–479. 10.1078/072320203322497518 14529191

[B10] Carrau F. Gaggero C. Aguilar P. S. (2015). Yeast diversity and native vigor for flavor phenotypes. *Trends Biotechnol.* 33 148–154. 10.1016/j.tibtech.2014.12.009 25630239

[B11] Cooper T. G. (1982). “Nitrogen metabolism in *Saccharomyces cerevisiae*,” in *The Molecular Biology of the Yeast Saccharomyces: Metabolism and Gene Expression*, ed. Strathern J. N. (Cold Spring Harbor, NY: Cold Spring Harbor Laboratory Press), 39–99.

[B12] Crépin L. Nidelet T. Sanchez I. Dequin S. Camarasa C. (2012). Sequential use of nitrogen compounds by *Saccharomyces cerevisiae* during wine fermentation: a model based on kinetic and regulation characteristics of nitrogen permeases. *Appl. Environ. Microbiol.* 78 8102–8111. 10.1128/AEM.02294-12 22983966 PMC3485930

[B13] Crépin L. Sanchez I. Nidelet T. Dequin S. Camarasa C. (2014). Efficient ammonium uptake and mobilization of vacuolar arginine by *Saccharomyces cerevisiae* wine strains during wine fermentation. *Microb. Cell Fact.* 13 1–13. 10.1186/s12934-014-0109-0 25134990 PMC4244049

[B14] Curiel J. A. Morales P. Gonzalez R. Tronchoni J. (2017). Different non-*Saccharomyces* yeast species stimulate nutrient consumption in *S. cerevisiae* mixed cultures. *Front. Microbiol.* 8:2121. 10.3389/fmicb.2017.02121 29163412 PMC5671574

[B15] Deed N. K. Van Vuuren H. J. J. Gardner R. C. (2011). Effects of nitrogen catabolite repression and di-ammonium phosphate addition during wine fermentation by a commercial strain of *S. cerevisiae*. *Appl. Microbiol. Biotechnol.* 89 1537–1549. 10.1007/s00253-011-3084-y 21246356

[B16] Díaz-Montaño D. M. Favela-Torres E. Córdova J. (2010). Improvement of growth, fermentative efficiency and ethanol tolerance of *Kloeckera africana* during the fermentation of agave tequilana juice by addition of yeast extract. *J. Sci. Food Agric.* 90 321–328. 10.1002/jsfa.3820 20355049

[B17] Fleet G. H. (2008). Wine yeasts for the future. *FEMS Yeast Res.* 8 979–995. 10.1111/j.1567-1364.2008.00427.x 18793201

[B18] Forsberg H. Ljungdahl P. (2001). Sensors of extracellular nutrients in *Saccharomyces cerevisiae*. *Curr. Genet.* 40 91–109. 10.1007/s002940100244 11680826

[B19] Giorello F. M. Berna L. Greif G. Camesasca L. Salzman V. Medina K. (2014). genome sequence of the native apiculate wine yeast *Hanseniaspora vineae* T02/19AF. *Genome Announc.* 2:e00530-14. 10.1128/genomeA.00530-14 24874663 PMC4038898

[B20] Gobert A. Tourdot-Maréchal R. Morge C. Sparrow C. Liu Y. Quintanilla-Casas B. (2017). Non-*Saccharomyces* yeasts nitrogen source preferences: impact on sequential fermentation and wine volatile compounds profile. *Front. Microbiol.* 8:2175. 10.3389/fmicb.2017.02175 29163451 PMC5672154

[B21] Godard P. Urrestarazu A. Vissers S. Kontos K. Bontempi G. van Helden J. (2007). Effect of 21 different nitrogen sources on global gene expression in the yeast *Saccharomyces cerevisiae*. *Mol. Cell. Biol.* 27 3065–3086. 10.1128/MCB.01084-06 17308034 PMC1899933

[B22] Gómez-Alonso S. Hermosín-Gutiérrez I. García-Romero E. (2007). Simultaneous HPLC analysis of biogenic amines, amino acids, and ammonium ion as aminoenone derivatives in wine and beer samples. *J. Agric. Food Chem.* 55 608–613. 10.1021/jf062820m 17263449

[B23] Gonzalez R. Quirós M. Morales P. (2013). Yeast respiration of sugars by non-*Saccharomyces* yeast species: a promising and barely explored approach to lowering alcohol content of wines. *Trends Food Sci. Technol.* 29 55–61. 10.1016/j.tifs.2012.06.015

[B24] Gutiérrez A. Chiva R. Beltran G. Mas A. Guillamón J. M. (2013). Biomarkers for detecting nitrogen deficiency during alcoholic fermentation in different commercial wine yeast strains. *Food Microbiol.* 34 1–11. 10.1016/j.fm.2012.12.004 23498202

[B25] Hofman-Bang J. (1999). Nitrogen catabolite repression in *Saccharomyces cerevisiae*. *Mol. Biotechnol.* 12 35–73. 10.1385/MB:12:1:35 10554772

[B26] Jolly N. P. Varela C. Pretorius I. S. (2014). Not your ordinary yeast: Non-*Saccharomyces* yeasts in wine production uncovered. *FEMS Yeast Res.* 14 215–237. 10.1111/1567-1364.12111 24164726

[B27] Lleixà J. Martín V. Portillo Mdel C. Carrau F. Beltran G. Mas A. (2016). Comparison of fermentation and wines produced by inoculation of *Hanseniaspora vineae* and *Saccharomyces cerevisiae*. *Front. Microbiol.* 7:338. 10.3389/fmicb.2016.00338 27014252 PMC4792884

[B28] López M. C. Mateo J. J. Maicas S. (2015). Screening of β-glucosidase and β-xylosidase activities in four non-*Saccharomyces* yeast isolates. *J. Food Sci.* 80 C1696–C1704. 10.1111/1750-3841.12954 26126488

[B29] Magasanik B. Kaiser C. A. (2002). Nitrogen regulation in *Saccharomyces cerevisiae*. *Gene* 290 1–18. 10.1016/S0378-1119(02)00558-9 12062797

[B30] Martín V. (2016). *Hanseniaspora vineae: Caracterización y su uso en la Vinificación*. Ph.D. thesis, Universidas de la República, Montevideo.

[B31] Martin V. Giorello F. Fariña L. Minteguiaga M. Salzman V. Boido E. (2016). De novo synthesis of benzenoid compounds by the yeast *Hanseniaspora vineae* increases the flavor diversity of wines. *J. Agric. Food Chem.* 64 4574–4583. 10.1021/acs.jafc.5b05442 27193819

[B32] Martin V. Valera M. J. Medina K. Boido E. Carrau F. (2018). Oenological Impact of the *Hanseniaspora/Kloeckera* yeast genus on wines—a review. *Fermentation* 4:76 10.3390/fermentation4030076.9

[B33] Masneuf-Pomarede I. Bely M. Marullo P. Albertin W. (2016). The genetics of non-conventional wine yeasts: current knowledge and future challenges. *Front. Microbiol.* 6:1563. 10.3389/fmicb.2015.01563 26793188 PMC4707289

[B34] Medina K. Boido E. Dellacassa E. Carrau F. (2012). Growth of non-*Saccharomyces* yeasts affects nutrient availability for *Saccharomyces cerevisiae* during wine fermentation. *Int. J. Food Microbiol.* 157 245–250. 10.1016/j.ijfoodmicro.2012.05.012 22687186

[B35] Medina K. Boido E. Fariña L. Gioia O. Gomez M. E. Barquet M. (2013). Increased flavour diversity of chardonnay wines by spontaneous fermentation and co-fermentation with *Hanseniaspora vineae*. *Food Chem.* 141 2513–2521. 10.1016/j.foodchem.2013.04.056 23870989

[B36] Padilla B. Gil J. V. Manzanares P. (2016). Past and future of non-*Saccharomyces* yeasts: from spoilage microorganisms to biotechnological tools for improving wine aroma complexity. *Front. Microbiol.* 7:411. 10.3389/fmicb.2016.00411 27065975 PMC4814449

[B37] Regenberg B. Düring-Olsen L. Kielland-Brandt M. C. Holmberg S. (1999). Substrate specificity and gene expression of the amino-acid permeases in *Saccharomyces cerevisiae*. *Curr. Genet.* 36 317–328. 10.1007/s002940050506 10654085

[B38] Ribéreau-Gayon P. Glories Y. Maujean A. Dubourdieu D. (2006). *Handbook of Enology: The Microbiology of Wine and Vinifications*. Hoboken, NJ: Wiley 10.1002/0470010398

[B39] Rollero S. Bloem A. Ortiz-Julien A. Camarasa C. Divol B. (2018). Altered fermentation performances, growth, and metabolic footprints reveal competition for nutrients between yeast species inoculated in synthetic grape juice-like medium. *Front. Microbiol.* 9:196. 10.3389/fmicb.2018.00196 29487584 PMC5816954

[B40] Ståhlberg A. Elbing K. Andrade-Garda J. M. Sjögreen B. Forootan A. Kubista M. (2008). Multiway real-time PCR gene expression profiling in yeast *Saccharomyces cerevisiae* reveals altered transcriptional response of ADH -genes to glucose stimuli. *BMC Genomics* 9:170. 10.1186/1471-2164-9-170 18412983 PMC2335116

[B41] Taillandier P. Lai Q. P. Julien-Ortiz A. Brandam C. (2014). Interactions between *Torulaspora delbrueckii* and *Saccharomyces cerevisiae* in wine fermentation: influence of inoculation and nitrogen content. *World J. Microbiol. Biotechnol.* 30 1959–1967. 10.1007/s11274-014-1618-z 24500666

[B42] Ter Schure E. G. Van Riel N. A. W. Verrips C. T. (2000). The role of ammonia metabolism in nitrogen catabolite repression in *Saccharomyces cerevisiae*. *FEMS Microbiol. Rev.* 24 67–83. 10.1016/S0168-6445(99)00030-3 10640599

[B43] Tesnière C. Brice C. Blondin B. (2015). Responses of *Saccharomyces cerevisiae* to nitrogen starvation in wine alcoholic fermentation. *Appl. Microbiol. Biotechnol.* 99 7025–7034. 10.1007/s00253-015-6810-z 26201494

[B44] Varela C. (2016). The impact of non-*Saccharomyces* yeasts in the production of alcoholic beverages. *Appl. Microbiol. Biotechnol.* 100 9861–9874. 10.1007/s00253-016-7941-6 27787587

[B45] Viana F. Belloch C. Vallés S. Manzanares P. (2011). Monitoring a mixed starter of *Hanseniaspora vineae*-*Saccharomyces cerevisiae* in natural must: impact on 2-phenylethyl acetate production. *Int. J. Food Microbiol.* 151 235–240. 10.1016/j.ijfoodmicro.2011.09.005 21962939

