# Supplementary material for: Analysis of the NCR Mechanisms in Hanseniaspora vineae and Saccharomyces cerevisiae During Winemaking
Source: Front Genet. 2019 Jan 11;9:747. doi: 10.3389/fgene.2018.00747 (PMC6338192; doi:10.3389/fgene.2018.00747)

Supplementary Material

**Analysis of the NCR mechanisms in *Hanseniaspora vineae* and *Saccharomyces cerevisiae* during winemaking**

**Jessica Lleixà^1^, Valentina Martín^2^, Facundo Giorello^2^, Maria del Carmen Portillo^1^, Francisco Carrau^2^, Gemma Beltran^1*^ and Albert Mas^1^**

*** Correspondence:** Gemma Beltran, [gemma.beltran@urv.cat](mailto:gemma.beltran@urv.cat)

**Supplementary Table 1.** Synthetic must composition used in the present work.

**Synthetic must composition (140 mg N/L)**

1 L

Glucose 100 g

Fructose 100 g

Citric acid 5 g

Malic acid 0.500 g

Tartaric acid 3 g

KH_2_PO_4_  0.750 g

K_2_SO_4_ 0.500 g

MgSO_2_ 7 H_2_O 0.250 g

CaCl_2_ 2 H_2_O 0.155 g

NaCl 0.200 g

Nitrogen (140 mg N/L)

NH_4_Cl (56 mg N/L) 0.214 g

Amino acid solution (84 mg N/L) 4.78 mL

Oligo-elements solution 1 mL

Vitamins solution 10 mL

Anaerobic factors 1 mL

**Vitamins stock solution**

1 L

Myo-inositol 2 g

Pantothenate calcium 0.150 g

Thiamine hydrochloride 0.025 g

Nicotinic acid 0.200 g

Pyridoxine 0.025 g

Biotine 3 mL (from a stock of 100 mg/L)

**Oligo-elements stock solution**

1 L

MnSO_4_ H_2_O 4 g

ZnSO_4_ 7 H_2_O 4 g

CuSO_4_. 5 _H2_O 1 g

KI 1 g

CoCl_2_ 6 H_2_O 0.4 g

H_3_BO_3_  1 g

(NH_4_)_6_Mo_7_O_24_  1 g

**Anaerobic factors stock solution**

0.100 L

Ergosterol 1.5 g

Oleic acid 0.5 mL

Tween 80 50 mL

Ethanol (absolute) until 100 mL

**Supplementary Table 2.** Ammonium content and amino acid stock solution content expressed as g L^-1^ and the corresponding nitrogen and YAN concentration in synthetic must in mg N L^-1^.

| **Amino acid** | **g L^-1^*** | **mg N L^-1^** | **mg YAN/L** |
| --- | --- | --- | --- |
| Asp | 4.42 | 2.22 | 2.22 |
| Glu | 11.96 | 5.44 | 5.44 |
| Ser | 7.80 | 4.97 | 4.97 |
| Gln | 49.92 | 45.76 | 22.88 |
| His | 3.38 | 1.46 | 1.46 |
| Gly | 1.82 | 1.62 | 1.62 |
| Thr | 7.54 | 4.24 | 4.24 |
| Arg | 36.79 | 42.45 | 14.15 |
| Ala | 14.56 | 10.95 | 10.95 |
| Tyr | 1.95 | 0.72 | 0.72 |
| Cis | 2.08 | 1.15 | 1.15 |
| Val | 4.42 | 2.53 | 2.53 |
| Met | 3.12 | 1.40 | 1.40 |
| Trp | 17.42 | 5.71 | 5.71 |
| Phe | 3.77 | 1.53 | 1.53 |
| Ile | 3.25 | 1.66 | 1.66 |
| Leu | 4.81 | 2.46 | 2.46 |
| Lys | 1.69 | 1.55 | 0.77 |
| Pro | 59.93 | 0,00 | 0.00 |
| **Total aas** |  | 137.83 | 85.88 |
| **Ammonia (NH_4_Cl)** | 0.214 | 56.00 | 56.00 |
| **Total YAN** |  |  | 141.88 |
| **Total N** |  | 193.83 |  |

* To achieve a final concentration of 140 mg YAN/L (190 mg N/L), 4.78 mL of amino acid stock solution is added to 1 L of synthetic must.

**Supplementary Table 3.** Homology of NCR related proteins between *H. vineae* and *S. cerevisiae* EC1118. Genes highlighted in bold are the ones which expression has been analyzed in this study.

| Gene name | Systematic name | Pfam domain | Putative orthologous  in *H. vineae* | Aminoacidic similarity | Single copy gene in  *H. vineae* |
| --- | --- | --- | --- | --- | --- |
| ***AGP1*** | YCL025C | AA_permease | g1661.t1;g1666.t1 | 50.43 %; 50.77 % | no |
| ***GAP1*** | YKR039W | AA_permease | g4653.t1 | 67.06 % | no |
| ***MEP2*** | YNL142W | Ammonium_transp;Ammonium transporter AmtB-like domain | g3765.t1 | 60.82 % | yes |
| ***PUT2*** | YHR037W | Aldehyde dehydrogenase domain | g905.t1 | 67.50 % | yes |
| *GAT1* | YFL021W | GATA zinc finger; Fungal protein of unknown function | g3143.t1 | 51.38% | yes |
| *GLN3* | YER040W | GATA zinc finger | g1456.t1 | 36.64% | yes |
| *GZF3* | YJL110C | GATA zinc finger | g1991.t1 | 45.23% | yes |
| *DAL80** | YKR034W | - | - | - | - |

**DAL80* is not present in *H. vineae*

**Supplementary Table 4.** Gene expression of *AGP1*, *GAP1*, *MEP2* and *PUT2* during the first 48 h of fermentation. Gene expression (2^-ΔΔCt) of each gene at different time points was determined during the first 48 h, considering 4 h after inoculation as the reference time. The values are expressed as the mean Log_10_ relative gene expression. The resulting Log_10_ 2^-ΔΔCt values were statistically analyzed using ANOVA and Tukey’s post-test. Different letters indicate significant differences in gene expression within each strain, p < 0.05.

|  | Time (hours) | *AGP1* | *GAP1* | *MEP2* | *PUT2* |
| --- | --- | --- | --- | --- | --- |
| *H. vineae* T02/5AF | 8 | 0,037^a^±0,023 | -0,543^a^±0,146 | 0,080^a^±0,332 | -0,133^a^±0,152 |
|  | 12 | -0,557^a,b^±0,206 | -0,734^a^±0,016 | 0,080^a^±0,200 | -0,134^a^±0,016 |
|  | 16 | -0,456^a,b^±0,185 | -0,318^a^±0,197 | 0,039^a^±0,251 | 0,533^a,b^±0,403 |
|  | 20 | -0,506^a,b^±0,358 | -0,437^a^±0,308 | 0,235^a^±0,294 | 0,703^a,b^±0,338 |
|  | 24 | -0,782^a,b^±0,085 | 0,434^a,b^±0,076 | 0,573^a,b^±0,109 | 1,236^b,c^±0,018 |
|  | 30 | -1,191^b^±0,621 | 1,447^b,c^±0,661 | 0,914^a,b^±0,604 | 1,061^b,c^±0,126 |
|  | 36 | -1,019^a,b^±0,621 | 1,722^c^±0,371 | 1,183^a,b^±0,159 | 1,699^c^±0,116 |
|  | 48 | -0,297^a,b^±0,180 | 2,014^c^±0,106 | 1,474^b^±0,266 | 1,744^c^±0,177 |
| *H. vineae* T02/19AF | 8 | -0,452^a^±0,346 | -0,669^a^±0,033 | -0,535^a^±0,220 | -0,243^a^±0,130 |
|  | 12 | -0,515^a^±0,117 | -0,666^a^±0,063 | -0,629^a^±0,118 | -0,103^a^±0,019 |
|  | 16 | -0,521^a^±0,422 | -0,766^a^±0,012 | -0,526^a^±0,220 | 0,038^a,b^±0,436 |
|  | 20 | -0,602^a^±0,210 | -0,573^a^±0,245 | -0,477^a^±0,351 | 0,243^a,b^±0,036 |
|  | 24 | -0,746^a,b^±0,255 | 0,236^b^±0,022 | 0,271^a,b^±0,053 | 0,471^a,b,c^±0,051 |
|  | 30 | -1,437^a,b^ ±0,238 | 0,560^a,b^±0,173 | 0,870^b^±0,022 | 0,716^b,c^±0,019 |
|  | 36 | -1,762^b^±0,131 | 0,642^a,b^±0,102 | 0,524^b^±0,431 | 1,105^c^±0,308 |
|  | 48 | -1,269^a,b^±0,201 | 0,738^a^±0,011 | 0,484^b^±0,066 | 1,111^c^±0,099 |
| *S. cerevisiae* QA23 | 8 | \| 0,106^a^±0,040 \| \| --- \| | \| -0,887^a^±0,224 \| \| --- \| | -0,330^a^±0,177 | -0,227^a^±0,097 |
|  | 12 | -0,018^a,b^±0,003 | -0,737^a^±0,437 | -0,442^a^±0,645 | -0,014^a^±0,321 |
|  | 16 | -0,541^a,b,c^±0,116 | 0,086^a,b^±0,099 | -0,149^a^±0,522 | 0,108^a^±0,519 |
|  | 20 | -0,593^a,b,c^±0,010 | 0,210^b,c^±0,077 | 0,146^a,b^±0,183 | 0,031^a^±0,044 |
|  | 24 | -0,061^a,b^±0,223 | 0,590^b,c^±0,188 | 0,513^a,b^±0,241 | 0,797^a,b^±0,329 |
|  | 30 | -1,022^c^±0,089 | 0,962^c,d^±0,100 | 0,565^a,b^ ±0,022 | 1,603^b,c^±0,036 |
|  | 36 | -0,705^b,c^±0,309 | 1,098^c,d^±0,162 | 0,809^a,b^ ±0,252 | 1,846^c^±0,171 |
|  | 48 | -0,370^a,b,c^±0,293 | 1,567^d^±0,282 | 1,385^b^±0,204 | 1,922^c^±0,150 |


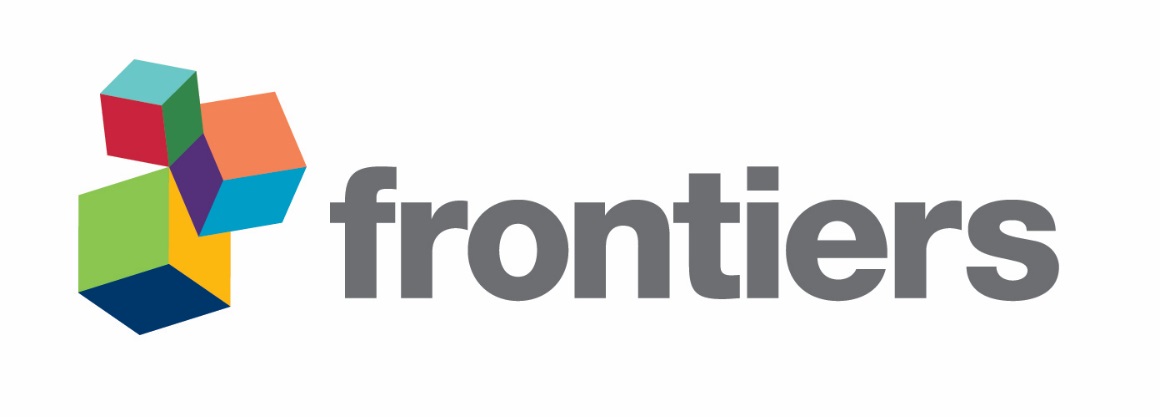

Supplement: Supplementary file 1 [file Data_Sheet_1.docx]
